# Photodynamic Activation of Cholecystokinin 1 Receptor with Different Genetically Encoded Protein Photosensitizers and from Varied Subcellular Sites

**DOI:** 10.3390/biom10101423

**Published:** 2020-10-08

**Authors:** Yuan Li, Zong Jie Cui

**Affiliations:** Institute of Cell Biology, Beijing Normal University, Beijing 100875, China; liyuanswkx@163.com

**Keywords:** genetically encoded protein photosensitizers (GEPP), cholecystokinin 1 receptor (CCK1R), singlet oxygen (^1^O_2_), calcium oscillations, KillerRed, miniSOG, miniSOG2, SOPP/miniSOG^Q103L^, Mr4511^C71G^, DsFbFP

## Abstract

Cholecystokinin 1 receptor (CCK1R) is activated by singlet oxygen (^1^O_2_) generated in photodynamic action with sulphonated aluminum phthalocyanine (SALPC) or genetically encoded protein photosensitizer (GEPP) KillerRed or mini singlet oxygen generator (miniSOG). A large number of GEPP with varied ^1^O_2_ quantum yields have appeared recently; therefore, in the present work, the efficacy of different GEPP to photodynamically activate CCK1R was examined, as monitored by Fura-2 calcium imaging. KillerRed, miniSOG, miniSOG2, singlet oxygen protein photosensitizer (SOPP), flavin-binding fluorescent protein from *Methylobacterium radiotolerans* with point mutation C71G (Mr4511^C71G^), and flavin-binding fluorescent protein from *Dinoroseobacter shibae* (DsFbFP) were expressed at the plasma membrane (PM) in AR4-2J cells, which express endogenous CCK1R. Light irradiation (KillerRed: white light 85.3 mW‧cm^−2^, 4’ and all others: LED 450 nm, 85 mW·cm^−2^, 1.5′) of GEPP_PM_-expressing AR4-2J was found to all trigger persistent calcium oscillations, a hallmark of permanent photodynamic CCK1R activation; DsFbFP was the least effective, due to poor expression. miniSOG was targeted to PM, mitochondria (MT) or lysosomes (LS) in AR4-2J in parallel experiments; LED light irradiation was found to all induce persistent calcium oscillations. In miniSOG_PM_-AR4-2J cells, light emitting diode (LED) light irradiation-induced calcium oscillations were readily inhibited by CCK1R antagonist devazepide 2 nM; miniSOG_MT_-AR4-2J cells were less susceptible, but miniSOG_LS_-AR4-2J cells were not inhibited. In conclusion, different GEPP_PM_ could all photodynamically activate CCK1R. Intracellular GEPP photodynamic action may prove particularly suited to study intracellular GPCR.

## 1. Introduction

Cholecystokinin 1 receptor (CCK1R) is expressed prominently in highly restricted brain regions, such as the basal ganglia, hippocampus, thalamus, hypothalamus, medulla oblongata [1,2,3,4,5,6,7,8,9], in the dorsal horn of the spinal cord [7,8], and in the peripheral ganglion and enteric neurons [10,11,12,13,14,15,16]. CCK1R plays important roles in central nervous system (CNS) functions, such as anxiety [17,18], appetite [19], brain development [1,20], and learning and memory [21]. CCK1R in the dorsal horn of the spinal cord [7,8] and in sensory ganglion neurons is essential in the transmission of peripheral satiety signals to the brain stem and higher up [13,22,23]. Activated peripheral CCK1R triggers gallbladder contraction [24] and pancreatic digestive enzyme secretion [24,25] but inhibits gastric acid secretion [26,27] and modulates large intestine motility [28]. 

CCK1R is unique among A class G protein-coupled receptors (GPCR) in that it is permanently activated ligand-independently by the lowest lying excited state molecular oxygen, the delta singlet oxygen (Δ^1^O_2_ or ^1^O_2_), usually generated in type II photodynamic action with chemical photosensitizer sulphonated aluminum phthalocyanine (SALPC) after a brief cellular incubation [29,30] in freshly isolated rat pancreatic acini or with genetically encoded protein photosensitizer (GEPP) KillerRed or mini singlet oxygen generator (miniSOG) target-expressed at the plasma membrane (PM) in rat pancreatic acinar tumor cell AR4-2J [31,32]. 

A typical photodynamic action involves three elements: light, a light-absorbing molecule (photosensitizer), and molecular oxygen. After absorption of a photon of a certain wavelength by a photosensitizer, the excited state photosensitizer molecule eventually undergoes either electron transfer or the energy transfer process. Electron transfer leads to the production of oxygen radicals (type I), such as superoxide anion (O_2_^−.^). Energy transfer to ground state molecular oxygen results in the production of ^1^O_2_ (type II) [29,30,31]. The absorption spectrum of the photosensitizer molecule determines the most effective wavelength of the irradiation light. The photophysicochemical property of the photosensitizer determines the ^1^O_2_ quantum yield (ϕ^1^O_2_) in type II photodynamic action. In contrast to chemical photosensitizers such as porphyrins and phthalocyanines, genetic manipulations will ensure that GEPP could be targeted to specified cell types or subcellular organelles with high precision. 

KillerRed is the first major GEPP to emerge, initially thought to generate solely superoxide anion (O_2_^−.^) [33,34,35] but has since been reported to probably generate ^1^O_2_ also [31,36], and later measured to have a ^1^O_2_ quantum yield (ϕΔ^1^O_2_ 0.008) of more than eight-fold higher than O_2_^−.^ [37]. The miniSOG was originally designed to correlate light and electron microscopy [38,39] but has since been used for many other purposes, such as acute modulation of the neurotransmitter release [40,41], modulation of the HCN2 channel function [42], and for many other delicate cellular and subcellular maneuverings [43]. 

Different variants of KillerRed and miniSOG have also appeared, either to monomerize the KillerRed dimer (SuperNova and GreenSuperNova) [44,45], to blue-shift the KillerRed excitation peak (GreenSuperNova and KillerOrange) [45,46,47], or for graded increases in miniSOG ^1^O_2_ quantum yields (miniSOG2; miniSOG^Q103L^; or singlet oxygen protein photosensitizer—SOPP, SOPP2, or SOPP3) [38,48,49,50,51,52], for enhanced miniSOG photostability after tethered dimerization with a more stable monomer (phiSOG and phiSOG^Q103V^) [53]. A red fluorescent protein (TagRFP) for tagging larger proteins has been found to show a noted ^1^O_2_ quantum yield [54]. ^1^O_2_-generating flavin-binding fluorescent protein (FbFP) photosensitizers originally from other source organisms, such as Pp2FbFP (from *Pseudomonas putida*), DsFbFp (from *Dinoroseobacter shibae*), EcFbFP (from *Bacillus subtilis*), CreiLOV (from *Chlamydomonas reinhardtii*), Mr4511^C71G^ (from *Methylobacterium radiotolerans*), and AsLOV2 (from *Aveva sativa*), have also appeared, all with desirably sufficient ^1^O_2_ quantum yields [55,56,57,58,59] (for a list of all GEPP that have appeared in the literature so far, see Table A1). 

In view of the above developments, it has become pertinent to examine whether the newly emerged GEPP could also be used for photodynamic CCK1R activation. Further, numerous works have reported that G protein-coupled receptors (GPCR) function not only from PM but, also, from intracellular membranes [60,61]. The role of intracellular miniSOG photodynamic action on CCK1R therefore also needs to be examined. Such works would potentially provide novel platforms upon which to elucidate the in vivo CCK1R function with high temporal and spatial precision. 

In the present work, it was found that plasma membrane (PM)-expressed KillerRed, miniSOG, miniSOG2, SOPP, Mr4511^C71G^, and DsFbFP in AR4-2J cells after light irradiation all photodynamically activated endogenous CCK1R in the rat pancreatic acinar tumor cell AR4-2J, triggering persistent calcium oscillations, with DsFbFP being the least effective. Interestingly, light irradiation of AR4-2J cells with miniSOG expressed at the plasma membrane (PM), mitochondria (MT), or lysosomes (LS) was found to trigger similarly persistent calcium oscillations, which were inhibited by CCK1R antagonist devazepide 2 nM with a graded sensitivity of PM > MT > LS. Therefore, GEPP expressed either at PM or intracellularly are both effective to photodynamically activate CCK1R, suggesting that photodynamic action may be particularly suited for the study of intracellular GPCR without the need for extracellularly added agonists to overcome multiple diffusion barriers. 

## 2. Materials and Methods

### 2.1. Materials

Sulfated cholecystokinin octapeptide (CCK) and CCK1R antagonist devazepide were from Tocris Cookson (Bristol, UK). Dulbecco’s modified Eagle’s medium (DMEM)/F12 medium was bought from Invitrogen (Shanghai, China). Fura-2 AM was from AAT Bioquest (Sunnyvale, CA, USA). JetPRIME transfection reagent was from PolyPlus-transfection SA (New York, NY, USA). Fetal bovine serum (FBS) was from Thermo Scientific (Shanghai, China). pKillerRed_PM_ vector was bought from Evrogen (Moscow, Russia). Ampicillin and kanamycin were from CWBio (Beijing, China). Endotoxin-free plasmid extraction kit and DH5à competent cells were from TianGen Biochemicals (Beijing, China). MitoTracker™ Red FM was from Invitrogen (Carlsbad, CA, USA). LysoTracker Red was from Beyotime (Shanghai, China). 

### 2.2. Cell Culture (AR4-2J, Escherichia coli)

AR4-2J was bought from The American Type Culture Collection (ATCC, Rockville, MD, USA) and cultured in DMEM/F12 supplemented with 20% fetal bovine serum in a CO_2_ incubator under humidified atmosphere (5% CO_2_/95% air) at 37 °C, as reported previously [32,62,63,64]. 

Solid *E. coli* medium LB/kana and LB/amp were sterilized and culture plates made. Liquid *E. coli* medium LB/kana and LB/amp had the same composition but without agar. 

### 2.3. Vector Constructs

Plasmid *pKillerRed_PM_* was bought from Evrogen (Moscow, Russia), proliferated in and harvested from competent *E. coli*. A mammalian codon-optimized *miniSOG* gene (GenBank accession number JX999997) was synthesized de novo from nucleotides at Genscript (Nanjing, China) with the following full sequence: ATGGAAAAGAGCTTTGTGATTACCGATCCGCGCCTGCCAGACAACCCGATCATTTTCGCGAGCGATGGCTTTCTGGAGTTAACCGAATATTCTCGTGAGGAAATTCTGGGTCGCAATGGCCGTTTCTTGCAGGGTCCGGAAACGGATCAAGCCACCGTGCAGAAAATCCGCGATGCGATTCGTGACCAACGCGAAATCACCGTTCAGCTGATTAACTATACGAAAAGCGGCAAGAAATTTTGGAACTTACTGCATCTGCAACCGATGCGCGATCAGAAAGGCGAATTGCAATATTTCATTGGTGTGCAGCTGGATGGCTAG. This synthesized full *miniSOG* gene sequence was inserted into plasmid *pKillerRed_PM_* (Evrogen, Moscow, Russia) to replace the *KillerRed* sequence. Competent *E. coli* were infected with the recombinant plasmid, cultured on solid LB/kana. Bacteria colonies were picked and further cultured in liquid LB/kana with shaking overnight. Proliferated plasmid was extracted with sequence verification. The plasmid so obtained was named *pminiSOG_PM_* due to the presence of the PM-localization sequence in the original Evrogen plasmid. After transfection with plasmid *pKillerRed_PM_* or *pminiSOG_PM_*, positive expressing AR4-2J cells were named KillerRed_PM_- or miniSOG_PM_-AR4-2J cells, as reported before [31,32]. 

For the construction of plasmid *pminiSOG2_PM_*, the *miniSOG2* [48] gene was synthesized de novo after rat codon optimization. The *miniSOG* sequence in plasmid *pminiSOG_PM_* was replaced with the synthesized *miniSOG2* sequence to obtain plasmid *pminiSOG2_PM_* (Genscript, Nanjing, China). The *miniSOG2* sequence was: ATGGAGAAGAGCTTCGTGATCACCGACCCCCGCCTGCCTGACAACCCAATCATCTTCGCCAGCGACTCCTTCCTGGAGCTGACCGAGTACTCCAGGGAGGAGATCCTGGGAAGGAACCCACGGTTCCTGAGAGGACCTGAGACCGACCAGGCAACCGTGCAGAAGATCCACGACGCCATCCGCGACCAGAGGGAGATCACCGTGCAGCTGATCAACTACACCAAGAGCGGCAAGAAGTTCTGGAACCTGTTCCGGCTGCAGCCAATCAGAGACCAGAAGGGCGAGCTGCAGTACTTCATCGGCGTGCAGCTGGACGGCTAA. AR4-2J cells transfected with plasmid *pminiSOG2_PM_* were named miniSOG2_PM_-AR4-2J cells. 

For the construction of plasmid *pSOPP_PM_*, the SOPP amino acid sequence [50] was used. The *SOPP* gene was synthesized de novo after rat codon optimization. The *miniSOG* sequence in plasmid *pminiSOG_PM_* was replaced to obtain plasmid *pSOPP_PM_* (Genscript, Nanjing, China), where the *SOPP* gene sequence was: ATGGAGAAGAGCTTCGTGATCACCGACCCCAGGCTGCCTGACAACCCAATCATCTTCGCCAGCGACGGCTTCCTGGAGCTGACCGAGTACTCCAGGGAGGAGATCCTGGGAAGGAACGGCCGGTTCCTGCAGGGACCCGAGACCGACCAGGCCACCGTGCAGAAGATCAGAGACGCCATCAGAGACCAGCGCGAGATCACCGTGCAGCTGATCAACTACACCAAGTCCGGCAAGAAGTTCTGGAACCTGCTGCACCTGCAGCCCATGCGGGACCAGAAGGGCGAGCTGCAGTACTTCATCGGCGTGCTGCTGGACGGCTAA. AR4-2J cells transfected with plasmid *pSOPP_PM_* as verified were named SOPP_PM_-AR4-2J cells.

For the construction of plasmid *pMr5411^C71G^_PM_*, the Mr5411^C71G^ protein sequence from [57] was used. The *Mr5411^C71G^* gene was synthesized de novo with rat codon optimization, which was used to replace the *miniSOG* sequence in *pminiSOG_PM_* (Genscript, Nanjing, China). The *Mr5411^C71G^* sequence was ATGGAGACCGGAGGAACCGCCACCAGCCACGTGCCAGACGAGCTGAAGGCAGAGTCCCACAGAGGCGACCCTTTCGCCGCAGCCGTGAGGGCAACCAGGATGCCCATGATCATCACCGACCCTGCCCAGCACGACAACCCAATCGTGTTCGTGAACGACGCCTTCCTGAAGCTGACCGGCTACACCAGGATGGAGGTGGTGGGAAGAAACGGCCGCTTCCTGCAGGGACCAGACACCGAGGCAGCAGCAGTGGACAGACTGAGGGCAGCCATCAGGCGGGAGGAGGACATCAGAGTGGACCTGCTGAACTACCGCAAGGACGGCAGCACCTTCCAGAACGCCCTGTACGTGGGACCCGTGAGGGACGAGGCAGGACGGGTGGTGTACTTCTTCGCCAGCCAGCTGGACGTGTCCGAGCACTACGCCCTGACCGCAGAGATCGAGAGGCTGAAGGCCGCCCTGGCCGAGGCCGAGGCCAAGCTGGCCGCCCGGTAG. AR4-2J cells transfected with plasmid *pMr5411^C71G^_PM_* were named *Mr5411^C71G^_PM_*-AR4-2J cells.

The *DsFbFP* gene [65] was synthesized de novo after mammalian codon optimization, which was then used to replace *miniSOG* from plasmid *pminiSOG_PM_* (Genscript, Nanjing, China). The *DsFbFP* gene sequence was ATGAGGCGGCACTACCGCGACCTGATCAGGAACACCCCCATGCCTGACACCCCACAGGACATCGCAGACCTGCGCGCCCTGCTGGACGAGGACGAGGCCGAGATGAGCGTGGTGTTCAGCGACCCATCCCAGCCCGACAACCCTATGATCTACGTGTCCGACGCCTTCCTGGTGCAGACCGGATACACCCTGGAGGAGGTGCTGGGAAGGAACGCAAGATTCCTGCAGGGACCAGACACCAACCCACACGCAGTGGAGGCAATCAGGCAGGGCCTGAAGGCAGAGACCAGATTCACCATCGACATCCTGAACTACAGGAAGGACGGCAGCGCCTTCGTGAACAGACTGCGCATCAGGCCTATCTACGACCCAGAGGGCAACCTGATGTTCTTCGCCGGCGCCCAGAACCCCGTGCTGGAGTAG. Positive AR4-2J cells after transfection with plasmid *pDsFbFP_PM_* were named DsFbFP_PM_-AR4-2J cells.

The plasmid *pminiSOG_MT_* was prepared by replacing the PM-localizing sequence ATGCTGTGCTGTATGAGAAGAACCAAACAGGTTGAAAAGAATGATGAGGACCAAAAGATC in *pminiSOG_PM_* with the mitochondrial (MT)-targeting sequence (MTS: ATGTCCGTCCTGACGCCGCTGCTGCTGCGGGGCTTGACAGGCTCGGCCCGGCGGCTCCCAGTGCCGCGCGCCAAGATCCATTCGTTGGGGGATCCACCGGTCGCCACC) (Genscript, Nanjing, China). *pminiSOG_LS_* was prepared by replacing the PM-localizing ATGCTGTGCTGTATGAGAAGAACCAAACAGGTTGAAAAGAATGATGAGGACCAAAAGATC sequence in *pminiSOG_PM_* with the lysosomal (LS) sequence (LTS: ATGAAGGGACAGGGAAGCATGGACGAGGGAACCGCCGACGAGAGGGCCCCCCTGATCCGGACC). Competent *E. coli* were infected with the plasmid, further cultured on solid LB/kana. Bacteria colonies were picked and cultured in liquid LB/kana with shaking overnight. Propagated plasmids were extracted for sequence verification. The plasmid constructs were designated *pminiSOG_MT_* and *pminiSOG_LS_*; transformed AR4-2J cells were named miniSOG_MT_-AR4-2J and miniSOG_LS_-AR4-2J cells, accordingly.

### 2.4. Transduction of AR4-2J Cells

AR4-2J cells were cultured in 6-well plates with one round glass cover-slip in each well, and to be transfected, cells were allowed to grow to 50–70% confluence. Plasmid (2 μg/well) and JetPRIME transfection reagent (4 μL/well) in JetPRIME buffer (200 μL) were added; then, AR4-2J cells were cultured for a further 24 h. Positive cellular GEPP expression was verified by confocal imaging of GEPP fluorescence: λ_ex_ 543 nm for KillerRed_PM_, and λ_ex_ 488 nm for miniSOG_PM_, miniSOG2_PM_, Mr4511^C71G^_PM_, and DsFbFP_PM_. Mitochondrial or lysosomal expressions of miniSOG (λ_ex_ 488 nm) were verified by colocalization with MitoTracker Red or LysoTracker Red (λ_ex_ 543 nm) in confocal imaging (Zeiss LSM510 META), objective × 60 oil. 

### 2.5. RT-PCR to Detect DsFbFP mRNA Expression

RT-PCR (reverse transcription-polymerase chain reaction): HiPure Total RNA Plus Mini Kit (Magen, Guangzhou, China) was used as instructed in the manufacturer’s manual for RNA extraction from AR4-2J and DsFbFP_PM_-AR4-2J cells. AR4-2J cells cultured in a Petri dish (35 mm) were transfected; twenty-four hours later, cells were washed in PBS before the extraction of RNA. RNA concentration was determined with a Nanodrop2000 nanospectrometer (Thermo Fisher Scientific, Wilmington, DE, USA). mRNA was reverse-transcribed with a GoScript Reverse Transcription Kit A5001 (Promega, Shanghai, China) to obtain cDNA. To a PCR tube was added Oligo (dT) 1 μL, RNA 1 μg, 70 °C denaturation for 5 min, cooled on ice for 5 min, before the addition of the reaction buffer (×5) 5 μL, RNAase inhibitor 1 μL, M-MLV reverse transcriptase 1 μL, dNTP (10 mM) 1.25 μL, topped up with diethyl pyrocarbonate (DEPC)-treated water to 25 μL. Reverse transcription conditions: 40 °C, 60 min and 70 °C, 15 min to obtain cDNA. PCR reaction: 2-μL cDNA template, 15-μL 2×Taq Master Mix (Vazyme, Nanjing, China), primers 1 μL each, topped up to 30 μL. Initial de-naturation 95 °C, 5 min; PCR cycles: 94 °C, 30 s, 60 °C, 30 s, 72 °C, 1.5 min, 30 cycles, and final prolongation 72 °C, 5 min. The RT-PCR product was run on 1% agarose gel with 0.01% GoodView (SaiBaiSheng Inc., Beijing, China) added, 120 V, 40 min before imaging. PCR primers for *DsFbFP* were: forward 5′-GGCACTACCGCGACCTGATC-3′ and reverse 5′-CTACTCCAGCACGGGGTTCT-3′. Primers for internal reference *GAPDH* were: forward 5′-GTGGAGTCTACTGGCGTCTT-3′ and reverse 5′-CCAGGATGCCCTTTAGTG-3′.

### 2.6. Photodynamic Action 

KillerRed_PM_-AR4-2J cells were irradiated with white light (85.3 mW·cm^−2^, 4 min) from a halogen cold light source (MegaLight 100, Hoya-Schott, Sapporo, Hokkaido, Japan) equipped with a condenser (HLL201). AR4-2J cells expressing PM-localizing miniSOG, miniSOG2, SOPP/miniSOG^Q103L^, Mr4511^C71^^G^, DsFbFP, MT- or LS-localizing miniSOG were irradiated with blue LED (450 nM, 85 mW·cm^−2^, 1.5 min) (LAMPLIC, Shenzhen, China). Power density was measured at the level of attached cells in the Sykes-Moore perfusion chamber with a power meter (IL1700, International Light Inc., Newburyport, MA, USA). Light-responding transfected AR4-2J cells were identified as GEPP-positive cells. 

### 2.7. Calcium Measurements

Parental control or transfected AR4-2J cells grown on glass cover-slips in 6-well plates were loaded with Fura-2 AM (final concentration 10 μM) for 1 h after assembly in the Sykes-Moore perfusion chamber. Cytosolic calcium was measured in an inverted fluorescent microscope (Nikon TE-2000U) (Shanghai, China) coupled to a Photon Technology International (PTI Inc., now HORIBA, Edison, NJ, USA) calcium measurement system with alternating excitations at 340 nm/380 nm (DeltaRam X); emitted Fura-2 fluorescence (dichroic mirror 400DCLP, emitter 510 ± 40 nm) was detected with a charge-coupled device (CCD) camera (NEO-5.5-CL-3, Andor/Oxford Instruments, Belfast, Northern Ireland, UK). Calcium concentration was expressed as Fura-2 fluorescence ratios F_340_/F_380_ and plotted against time with SigmaPlot (Palo Alto, CA, USA), as reported before [29,30,32,62,63,64,66]. In figures shown in Results, original colored calcium tracings were each from individual cells, all from 1 out of *N* (as indicated in figures, *N* ≥ 3) identical experiments.

### 2.8. Data Presentation and Statistical Analysis 

All calcium tracings and other graphs were plotted with SigmaPlot. For calculation and comparison of the strength of induced calcium responses, the calcium peak area above the baseline was integrated (usually per 10 min, unless stated otherwise). Statistical data from *N* (as indicated) independent experiments were presented in bar graphs as mean ± SEM, unless specifically stated otherwise. Student’s *t*-test was used for statistical analysis against controls, and *p* < 0.05 was taken as significant and indicated with an asterisk (*).

## 3. Results

### 3.1. PM-Delimited Photodynamic CCK1R Activation in AR4-2J Cells with GEPP KillerRed, miniSOG, miniSOG2, SOPP, Mr4511, and DsFbFP

**Figure 1:**
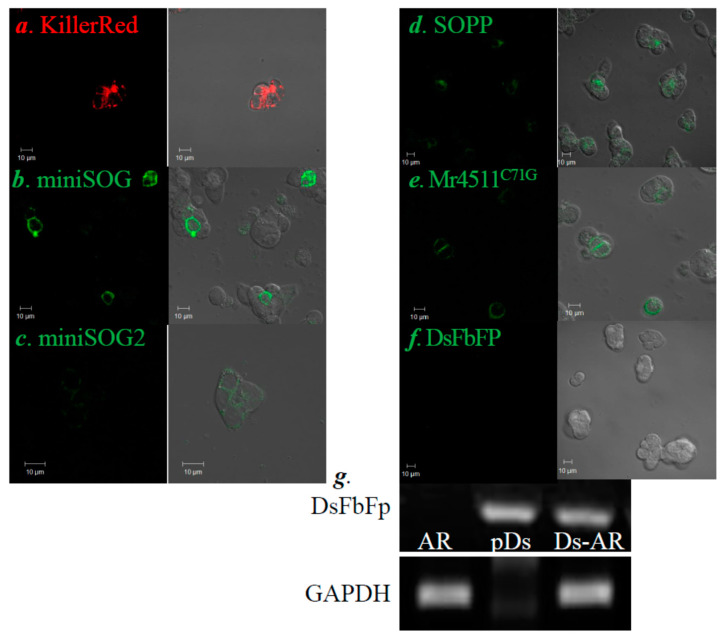
Plasma membrane (PM) expression of genetically encoded protein photosensitizers KillerRed, miniSOG, miniSOG2, SOPP, Mr4511^C71G^, and DsFbFP. (**a**–**f**) AR4-2J cells were transfected with plasmids *pKillerRed_PM_*, *pminiSOG_PM_*, *pminiSOG2_PM_*, *pSOPP_PM_*, *pMr4511^C71G^_PM_*, and *pDsFbFP_PM_*, respectively; twenty-four hours later, KillerRed (**a**, λ_ex_ 543 nm), miniSOG, miniSOG2, SOPP, Mr4511*^C71G^*, and DsFbFP (**b–f**, λ_ex_ 488 nm) fluorescence was confocal imaged (fluorescence and bright field-merged images shown). Scale bars: 10 μm. (**g**) Reverse transcript (RT)-PCR: AR, parental AR4-2J cells; Ds-AR, DsFbFP_PM-_AR4-2J cells; and *pDs*, plasmid *pDsFbFP_PM_* as the positive control. GAPDH was used as the internal reference.

Plasmid *pKillerRed_PM_*, *pminiSOG_PM_*, *pminiSOG2_PM_*, *pSOPP_PM_*, *pMr4511^C71G^_PM_*, and *pDsFbFP* (for the full protein sequence of each of these GEPP, see Table A2) were conducted into AR4-2J cells, and the protein expression was confirmed by GEPP fluorescence imaging (Figure 1a–f). In comparison with the other GEPP (KillerRed, miniSOG, miniSOG2, SOPP and Mr4511^C71G^), DsFbFP demonstrated markedly dimmer fluorescence, suggesting poorer protein expression, as shown in the confocal images (Figure 1f). RT-PCR experiments revealed that DsFbFP was abundantly expressed at the mRNA level after transfection with plasmid *pDsFbFP_PM_* (Figure 1g). The reason for the rather dim DsFbFP fluorescence will be further elaborated below and in Discussion. 

**Figure 2:**
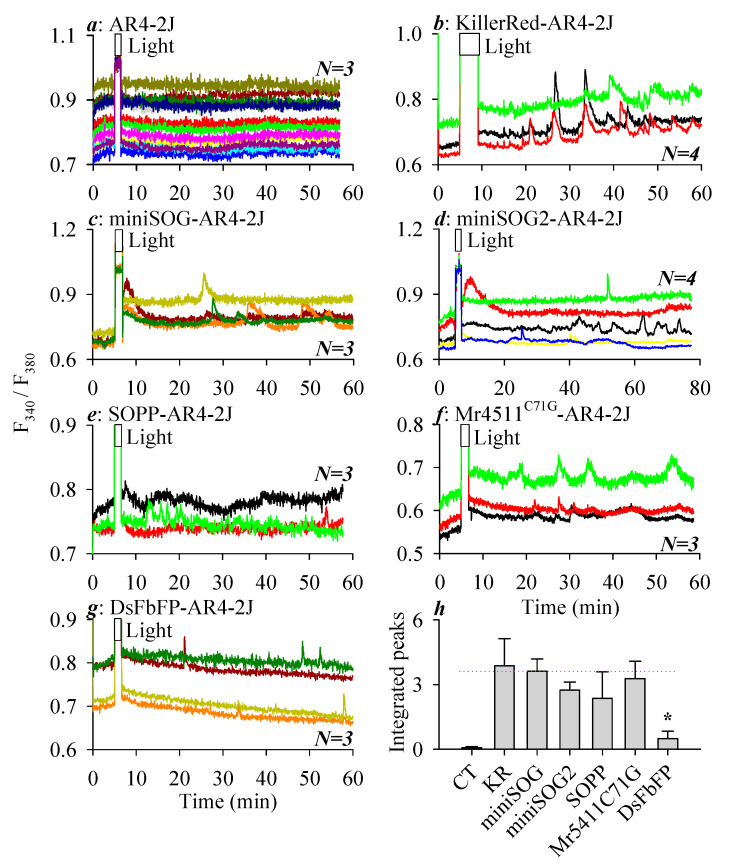
Photodynamic CCK1R activation with genetically encoded protein photosensitizers KillerRed, miniSOG, miniSOG2, SOPP, Mr4511, and DsFbFP. Fura-2 AM-loaded AR4-2J (**a**), KillerRed_PM_-AR4-2J (**b**), miniSOG_PM_-AR4-2J (**c**), miniSOG2_PM_-AR4-2J (**d**), SOPP_PM_-AR4-2J (**e**), Mr4511^C71G^_PM_ (**f**), and DsFbFP-AR4-2J cells (**g**) were perfused, and LED light (**a**, **c**–**g**, 450 nm, 85 mW·cm^−2^, 1.5 min) or white light (***b***, 85.3 mW‧cm^−2^, 4 min) was applied, as indicated by the horizontal bars. LED light (450 nm, 85 mW·cm^−2^, 1.5 min) irradiation of AR4-2J cells (**a**, *N* = *4*). White light irradiation (85.3 mW‧cm^−2^, 4 min) of KillerRed_PM_-AR4-2J cells (**b**, *N* = *4*). LED light (450 nm, 85 mW·cm^−2^, 1.5 min) irradiation of miniSOG_PM_-AR4-2J (***c****, N* = *3*), miniSOG2_PM_-AR4-2J (***d****, N* = *4*), SOPP_PM_-AR4-2J (**e**, *N* = *3*), Mr4511^C71G^_PM_-AR4-2J (**f**, *N* = *3*), or DsFbFP-AR4-2J (**g**, *N* = *3*) cells. The colored calcium tracings in (**a**–**g**) were each from individual cells from one of *N* (as indicated) identical experiments, ordinate being the fluorescence ratios in F_340_/F_380_ and abscissa being time in min, as indicated. Integrated calcium peaks (per 10 min) in calcium tracings as represented in (**a**–**g**) from *N* experiments were calculated and plotted as a bar graph (**h**). The thin, pink, dashed horizontal line in (**h**) indicates the level of miniSOG photodynamic effect. No statistical difference was found between miniSOG and KillerRed (KR) or miniSOG2, SOPP, Mr4511^C71G^, but the DsFbFP photodynamic effect was significantly less compared with miniSOG (*p* < 0.05), as indicated by an asterisk (^*^). CT: control—parental AR4-2J cells not transfected with any genetically encoded protein photosensitizer (GEPP).

The baseline calcium level remained flat after blue LED light irradiation (450 nm, 85 mW‧cm^−2^, 1.5 min) in parental AR4-2J cells (Figure 2a). Baseline calcium remained flat as well in KillerRed_PM_-, miniSOG_PM_-, miniSOG2_PM_-, SOPP_PM_-, Mr4511^C71G^_PM_-, and DsFbFP_PM_-AR4-2J cells without light irradiation (not shown). In parallel experiments, white light irradiation (85.3 mW‧cm^−2^, 4 min) from the halogen light source was found to induce sustained calcium oscillations in KillerRed_PM_-AR4-2J cells (Figure 2b). Similarly, blue LED light irradiation (450 nm, 85 mW‧cm^−2^, 1.5 min) elicited persistent calcium oscillations in miniSOG_PM_- (Figure 2c), miniSOG2_PM_- (Figure 2d), SOPP_PM_- (Figure 2e), Mr4511^C71G^_PM_- (Figure 2f), and DsFbFP_PM_-AR4-2J (Figure 2g) cells. Note the very sparse, but distinct, calcium spikes in DsFbFP_PM_-AR4-2J cells (Figure 2g). This weak calcium response combined with the dim fluorescent imaging shown in Figure 1 would suggest that DsFbFP indeed was conducted into AR4-2J cells, as verified by RT-PCR experiments (Figure 1g). A quantitative analysis of photodynamically-induced calcium spiking in each case found no statistically significant difference in photodynamic CCK1R activation with KillerRed, miniSOG, miniSOG2, SOPP, or Mr4511^C71G^ as the photosensitizer, but the effect was markedly diminished or weaker with DsFbFP_PM_ as the photosensitizer (*p* < 0.05, Figure 2h). 

### 3.2. miniSOG Photodynamic Action in AR4-2J Cells at PM, MT, or LS with Graded Sensivitivity towards Extracellular CCK1R Antagonist

**Figure 3:**
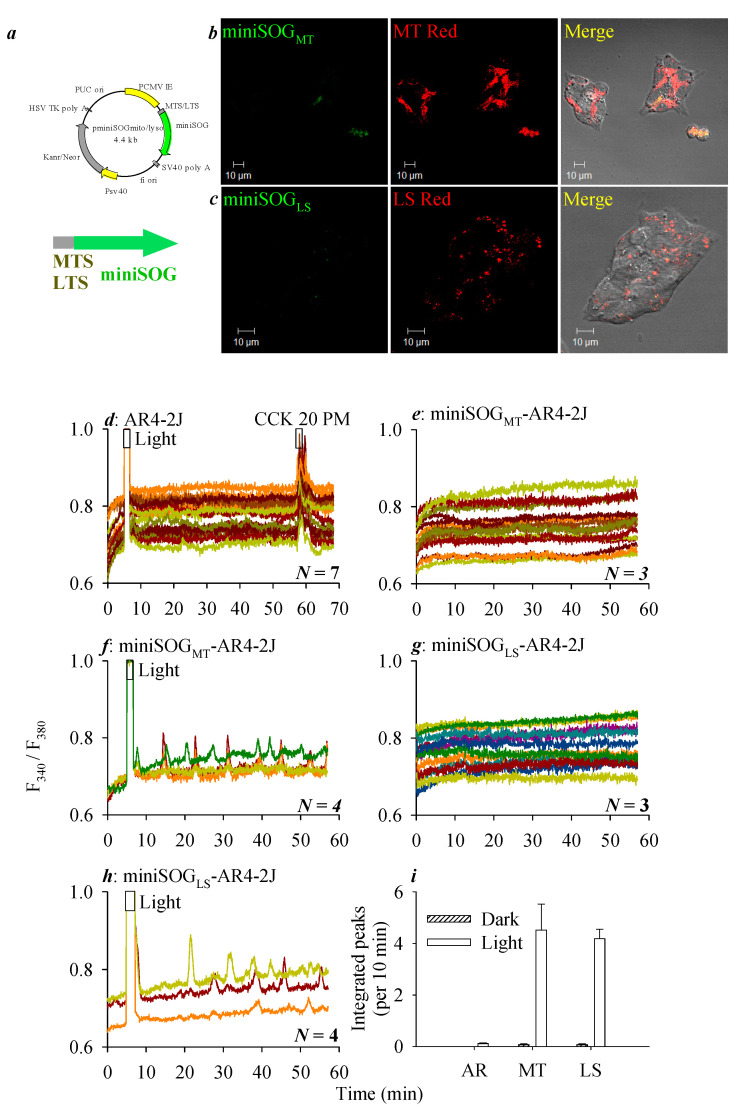
Targeted mitochondrial or lysosomal miniSOG photodynamic action induced persistent calcium oscillations in AR4-2J cells. (**a**) Plasmid *pminiSOG_MT_* or *pminiSOG_LS_* targeting mitochondria (MT) or lysosomes (LS). miniSOG_MT_ (**b**) or miniSOG_LS_ -AR4-2J cells (***c***) were confocal-imaged 24 h after transfection. miniSOG (λ_ex_ 488 nm) colocalization with MitoTracker^TM^ Red (**b**, λ_ex_ 543 nm) or LysoTracker Red (**c**, λ_ex_ 543 nm) were verified, as seen in the merged images. MT Red: MitoTracker Red and LT Red: LysoTracker Red. Scale bars: 10 μm. Fura-2 AM-loaded AR4-2J (**d**), miniSOG_MT_-AR4-2J (**e**,**f**), or miniSOG_LS_-AR4-2J cells (**g**,**h**) were perfused; CCK 20 pM or LED illumination (450 nm, 85 mW·cm^−2^, 1.5 min) were applied, as indicated by the horizontal bars. The colored calcium tracings each were from individual cells measured simultaneously from one of *N* (as indicated) identical experiments, ordinate being fluorescence ratios in F_340_/F_380_ and abscissa being time in min, as indicated. LED light irradiation had no effect on baseline calcium in parental AR4-2J cells (**d**). Note that, in the absence of LED light, the baseline calcium concentration remained stable in (**e**,**g**), but LED light induced persistent calcium oscillations both in miniSOG_MT_-AR4-2J (***f***) and miniSOG_LS_-AR4-2J cells (**h**). (**i**) Integrated calcium peaks (per 10 min) in calcium tracings as represented in (**d**–**h**) were calculated from *N* experiments. AR: AR4-2J cells as shown in (**d**). MT: miniSOG_MT_ -AR4-2J cells without (**e**) or with (**f**) light. LS: miniSOG_LS_-AR4-2J cells without (**g**) or with (**h**) LED light irradiation.

Other than targeting miniSOG to the plasma membrane (PM) like previously reported [31,32], we now examined the miniSOG photodynamic action from the mitochondria (MT) or lysosomes (LS). The miniSOG was targeted to the mitochondria or lysosomes by transfection with vectors *pminiSOG_MT_* and *pminiSOG_LS_* (Figure 3a), with a mitochondrial localization sequence from human cytochrome C oxidase subunit VIII (MSVLTPLLLRGLTGSARRLPVPRAKIHSLGDPPVAT) [67,68] or with a lysosomal localization sequence from the C-terminal tail sequence of lysosomal-associated membrane protein 1 (LIMP II: KGQGSMDEGTADERAPLIRT) (LTS), respectively [69]. Twenty-four (24) hours after the transfection of AR4-2J cells with plasmid *pminiSOG_MT_* or *pminiSOG_LS_*, confocal imaging confirmed miniSOG expression in the mitochondria or lysosomes, as judged by their respective co-localization with MitoTracker or LysoTracker (Figure 3b,c). 

Blue LED light irradiation (450 nm, 85 mW·cm^−2^, 1.5 min) was found to have no effect on the baseline calcium level in parental AR4-2J cells, but in these cells, CCK 20 pM induced marked calcium responses (Figure 3d). The baseline calcium remained stable in miniSOG_MT_-AR4-2J cells kept in the dark (Figure 3e), whereas LED light irradiation (450 nm, 85 mW·cm^−2^, 1.5 min) induced long-lasting calcium oscillations in miniSOG_MT_-AR4-2J cells (Figure 3f). The baseline calcium concentration also remained flat in non-irradiated miniSOG_LS_-AR4-2J cells (Figure 3g), but light irradiation (450 nm, 85 mW·cm^−2^, 1.5 min) similarly induced long-lasting calcium oscillations in miniSOG_LS_-AR4-2J cells (Figure 3h). 

Quantitative analysis of calcium responses in original tracings, as represented in Figure 3d–h, from *N* (3–7) identical experiments was calculated and plotted as bar graphs (i). Note the sharp difference in the calcium response after LED light irradiation in parental AR4-2J cells, and dark or light responses in MT or LS miniSOG-transfected AR4-2J cells (i). 

**Figure 4:**
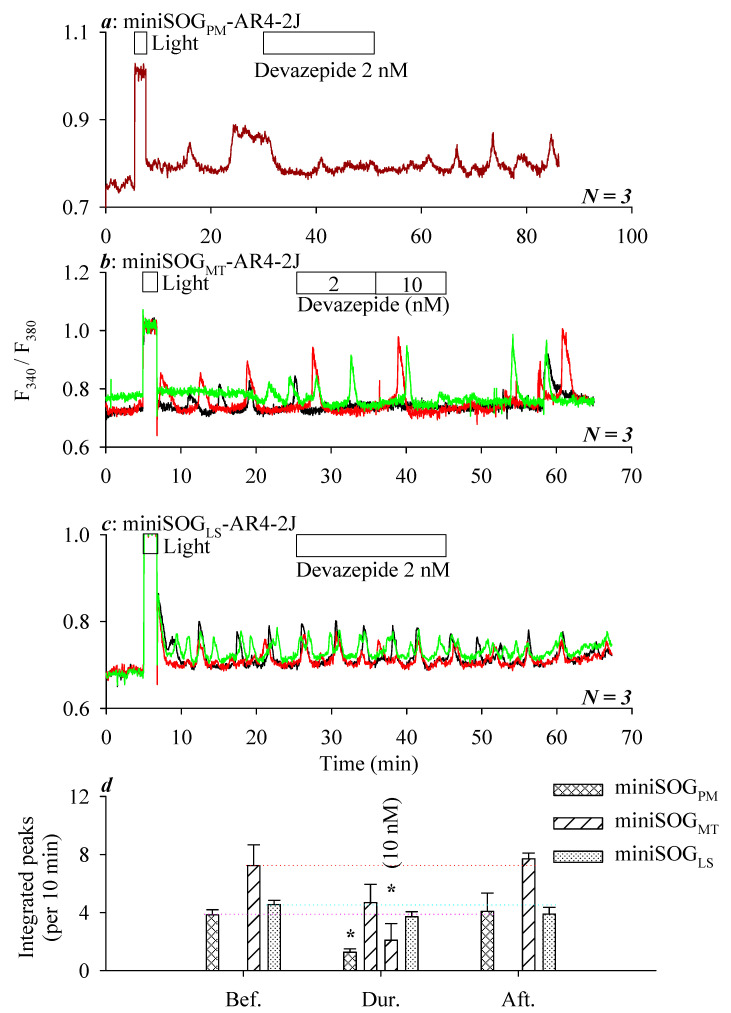
Calcium oscillations induced by miniSOG photodynamic action at the plasma membrane (PM), mitochondria (MT), or lysosomes (LS) in AR4-2J cells showed graded sensitivity to inhibition by the CCK1R antagonist devazepide at 2 nM: PM > MT > LS. Fura-2 AM-loaded miniSOG_PM_-AR4-2J (**a**), miniSOG_MT_-AR4-2J (**b**), or miniSOG_LS_-AR4-2J cells (**c**) were perfused; devazepide (2, 10 nM) and LED light (450 nm, 85 mW·cm^−2^, 1.5 min) were applied as indicated by the horizontal bars. The calcium tracings are each from individual cells measured simultaneously in one out of *N* (as indicated) identical experiments, the ordinate being fluorescence ratios in F_340_/F_380_ and the abscissa being time in min, as indicated. Integrated calcium peaks (per 10 min) from *N* experiments were plotted, and statistically significant differences between before (Bef.) and during (Dur.) or after (Aft.) devazepide application were indicated by an asterisk (*) at *p* < 0.05 (**d**), for the devazepide inhibition of miniSOG photodynamically induced calcium oscillations with miniSOG_PM_ or miniSOG_LS_. Note the thin pink (PM), cyan (MT), and red (LS) dotted horizontal lines (3) at the level before the devazepide application in miniSOG_PM_-AR4-2J, miniSOG_MT_-AR4-2J, and miniSOG_LS_-AR4-2J cells, respectively (**d**). In miniSOG_MT_-AR4-2J cells before devazepide 2 nM, the calcium response was 100%, during devazepide 2 nM, 65%, during devazepide 10 nM, 29%, and after devazepide, 106%. For better consistence, for miniSOG_MT_-AR4-2J cells, the statistical data in (**d**) were from all individual cells in one out of three identical experiments shown in (**b**).

In parallel experiments, it was found that calcium oscillations induced by LED light irradiation in miniSOG_PM_-AR4-2J cells (i.e., miniSOG_PM_ photodynamic action at the plasma membrane) were inhibited nearly completely by the CCK1R antagonist devazepide 2 nM (Figure 4a). LED light irradiation-triggered calcium oscillations were inhibited in miniSOG_MT_-AR4-2J cells by the CCK1R antagonist devazepide 2 nM but to a lesser extent; a complete blockade was seen with increased devazepide at 10 nM (Figure 4b). LED light irradiation-induced calcium oscillations in miniSOG_LS_-AR4-2J cells, however, were not inhibited by devazepide 2 nM (Figure 4c). 

Quantitative analysis confirmed a significant inhibition by devazepide 2 nM of miniSOG_PM_ photodynamic action (Bef.: 3.84 ± 0.35/100%, Dur.: 1.26 ± 0.23/33%, and Aft.: 4.08 ± 1.26/106%; *N* = 3) but no effect on miniSOG_LS_ photodynamic action (Bef.: 4.55 ± 0.29/100%, Dur.: 3.72 ± 0.34/82%, and Aft.: 3.89 ± 0.47/85%; *N* = 3) (Figure 4d). miniSOG_MT_ photodynamic action was inhibited without significance by devazepide 2 nM (Bef.: 6.11 ± 1.05/100%, Dur.: 3.98 ± 0.86/65%, and Aft. 6.33 ± 1.00/103%; *N* = 3, not shown in bar graph). For the representative experiment shown in Figure 4b, for example, miniSOG_MT_ photodynamic action was inhibited without significance by devazepide 2 nM (from 100% to 65%) but significantly by devazepide 10 nM (from 100% to 29%) (Figure 4d). 

Figure 4 suggests that calcium oscillations are triggered by miniSOG photodynamic action not only at the plasma membrane but, also, in mitochondria and lysosomes; a graded inhibition of the triggered calcium oscillations by CCK1R antagonist devazepide 2 nM was found, depending on the subcellular site of the miniSOG photodynamic action: PM > MT > LS.

## 4. Discussion

In the present work, it was found that PM-expressed KillerRed, miniSOG, miniSOG2, SOPP, Mr4511^C71G^, and DsFbFP after light irradiation all photodynamically activated CCK1R to induce persistent cytosolic calcium oscillations in AR4-2J cells, but the photodynamic effect of DsFbFP was much reduced in comparison, likely due to poor protein expression. Permanent photodynamic CCK1R activation was achieved in AR4-2J cells by miniSOG expression not only at the plasma membrane (PM) but, also, in mitochondria (MT) and lysosomes (LS). Calcium oscillations induced by miniSOG photodynamic action at intracellular sites showed reduced sensitivity to inhibition by CCK1R antagonist devazepide 2 nM with the order of PM > MT > LS. 

CCK1R is unique among A class GPCR in that it is activated permanently by ^1^O_2_ generated in type II photodynamic action with SALPC, KillerRed, or miniSOG as the photosensitizer [29,30,31,32]. In the present work, both KillerRed and miniSOG were target-expressed at the plasma membrane in AR4-2J cells (Figure 1); light irradiation (KillerRed with white light 85.3 mW‧cm^−2^, 4 min and miniSOG with blue LED 450 nm, 85 mW‧cm^−2^, 1.5 min) triggered long-lasting calcium oscillations in both KillerRed_PM_-AR4-2J and miniSOG_PM_-AR4-2J cells (Figure 2). Other than KillerRed and miniSOG, photodynamic CCK1R activation with miniSOG2, SOPP (miniSOG^Q103L^), Mr4511^C71G^, and DsFbFP expressed at the plasma membrane (Figure 1) were also found, similarly inducing persistent calcium oscillations in transfected AR4-2J cells (Figure 2). No significant difference in the calculated intensity of calcium oscillations due to photodynamic CCK1R activation was found among KillerRed, miniSOG, miniSOG2, SOPP, or Mr4511^C71G^, but the photodynamic efficacy of DsFbFP was markedly smaller (Figure 1; Figure 2). These data would suggest that, other than KillerRed and miniSOG, the newly emerged GEPP miniSOG2, SOPP/miniSOG^Q103L^, Mr4511^C71G^, and DsFbFP could also be used to permanently activate CCK1R photodynamically. 

Although the DsFbFP protein level was low, as shown by the dim DsFbFP fluorescence, DsFbFP mRNA were expressed at sufficient levels in AR4-2J cells, as verified by RT-PCR experiments (Figure 1g). Although the DsFbFP fluorescence quantum yield (Φ_Fluo_ = 0.35) [56] is the highest of all GEPP examined in the present work, DsFbFP is also known to be easily photobleached, photobleaching eight times faster than miniSOG (t_bl50%_ miniSOG: 2.85 min and DsFbFP: 0.35 min) [65]. This was borne out by the fact that, although we were able to observe with the naked eye in transfected AR4-2J cells moderate DsFbFp fluorescence under the confocal microscope, photobleaching by the scanning laser light was so fast that it was difficult to capture the fluorescence image. However, the photobleaching of DsFbFP by the excitation light (340, 380 nm) might not be as significant during Fura-2 calcium imaging; therefore, we were able to observe distinct calcium spikes in DsFbFP_PM_-AR4-2J cells after blue LED irradiation (Figure 2g). DsFbFP (from *Dinoroseobacter shibae*) has been known to be expressed in CHO-K1 cells as a dimer, which might also affect its fluorescence, unlike the monomeric miniSOG, miniSOG2, or SOPP, all sourced from *Arabidopsis thaliana* [65,70]. The weak DsFbFP fluorescence in transfected AR4-2J cells is also likely a reflection of poor protein translation from mRNA, since the DsFbFP photodynamic activation of CCK1R was much diminished in comparison with miniSOG (Figure 2). Additional code optimization for mammalian cell expression might improve DsFbFP protein expression. Alternatively, a heterodimeric construct similar to phiSOG [53] might facilitate DsFbFP fluorescence imaging or stabilize its photodynamic efficacy. 

miniSOG produces ^1^O_2_ after light irradiation in a type II photodynamic action [49,52,71], with an ϕ^1^O_2_ of 0.03 (as reviewed in [43]). KillerRed was originally thought to be primarily a type I photosensitizer to generate mainly superoxide anion (O_2_^−.^) [43], but more recently, it has been suggested that KillerRed in the cellular milieu almost certainly also generates ^1^O_2_ [31,36,43]. Photochemical measurements indeed revealed that KillerRed actually generates eight times more ^1^O_2_ than O_2_^−.^, with an ϕ^1^O_2_ of 0.008, whilst monomeric KillerRed SuperNova has an ϕ^1^O_2_ of 0.02 [37]. miniSOG2 is reported to be about eight times as efficient as miniSOG to produce reactive oxygen species [48]. The singlet oxygen protein photosensitizer (*SOPP* or miniSOG^Q10^^3L^) has a ϕ^1^O_2_ of 0.25 [50]. Mr4511^C71G^ sourced from *Methylobacterium radiotolerans* has a ϕ^1^O_2_ of 0.19 [57]. DsFbFP has the highest ϕ^1^O_2_ among the lot at 0.33 [56]. Although these protein photosensitizers have varied values of ϕ^1^O_2_ from 0.008 to 0.33 (see Table A1), no significant difference in their photodynamic activation of CCK1R (i.e., integrated calcium oscillations) was found except DsFbFP, which has the highest ϕ^1^O_2_ value of 0.33 but the weakest photodynamic efficacy (Figure 2). This indicates that photodynamic CCK1R activation could be achieved with GEPP with wide-ranging values of ϕ^1^O_2_. However, care must be taken with protein photosensitizers with higher values of ϕ^1^O_2_ to avoid photobleaching or possible activation by the ambient light during imaging or calcium measurements. 

It has been found by others that blue LED irradiation (450 nm, 0.8 W‧cm^−2^, 1 min) induced phototoxicity in a small percentage of cells two hours after light irradiation of miniSOG-HEK293, miniSOG2-HEK293, and SOPP-HEK293 cells, but no marked difference was found in HEK293 cells expressing SOPP (9%) with a ϕ^1^O_2_ of 0.25 or miniSOG (11%) with a ϕ^1^O_2_ of 0.03 [48]. The reason for the satisfactory performance by miniSOG with a ϕ^1^O_2_ of only 0.03 to permanently activated photodynamically CCK1R in miniSOG_PM_-AR4-2J cells in the present work might be related to the progressive photochemical transformation of the fluorophore flavin mononucleotide (FMN) to lumichrome and the photo-oxidization of internal residues in miniSOG to significantly increase its ϕ^1^O_2_ up to 10-fold [72]. In this regard, it is interesting to note that with AsLOV2 (not studied in the present work, and its ϕ^1^O_2_ is not determined; see Table A1), the LOV2 domain of *Avena sativa* phototropin 1, light irradiation was found to induce progressive photochemical dissociation or the release of FMN from the AsLOV2 protein moiety, leading to significantly increased ϕ^1^O_2_ [59]. 

Of the GEPP KillerRed, miniSOG, miniSOG2, SOPP, Mr4511^C71G^, and DsFbFP examined in the present work, KillerRed is excited by the red component (585 nm) of the visible spectrum (full-spectrum white light was used in the present work), whereas all others by blue light (450 nm) (Figure 2) (for the photophysical parameters of GEPP, see Table A2). Although the KillerRed structure is completely different from miniSOG and other flavin-binding protein photosensitizers, the most satisfactory performance by KillerRed reported in the present work (Figure 2) might warrant further photochemical studies to examine possible increases in ϕ^1^O_2_ during light irradiation. 

For possible future in vivo applications, KillerRed might be better-suited due to its excitation by red light, instead of by blue light for miniSOG, miniSOG2, SOPP, Mr4511^C71G^, and DsFbFP. It would be ideal if KillerRed could be subjected to further annotations, to shift its maximal excitation peak toward even longer wavelengths (a red shift), possibly by genetic code expansion [73], instead of the blue shifts observed in KillerOrange [46,47] and GreenSuperNova [45]. Although KillerRed is twice the size of miniSOG and larger than MR4511^C71G^, DsFbFP (see Table A2), KillerRed fusion to CCK1R (CCK1R-KillerRed) were found to still result in, after light irradiation, the effective photodynamic activation of the in-frame CCK1R [31]. In the future, efficient red light-powered KillerRed photodynamic action might be applied to modulate other vital protein machines, such as the membrane molecular architecture of zymogen granules [74]. 

**Figure 5:**
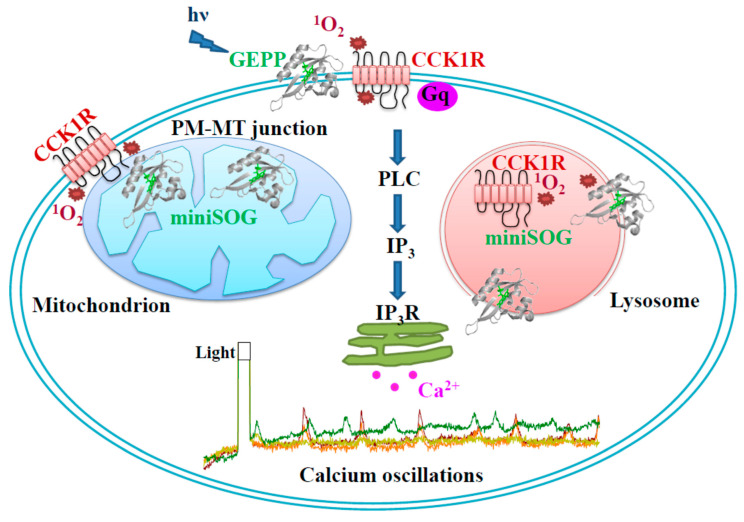
Plasma membrane (PM), mitochondrial (MT), and lysosomal (LS) miniSOG photodynamic actions induced calcium oscillations in AR4-2J cells. Plasma membrane (PM)-, mitochondria (MT)-, or lysosome (LS)-localized miniSOG upon blue LED light (hν) irradiation generates ^1^O_2_, which then activates CCK1R to trigger cytosolic calcium oscillations. In PM, miniSOG-generated ^1^O_2_ directly oxidizes CCK1R. In MT, miniSOG-generated ^1^O_2_ may diffuse to the PM via MT-PM contact sites, leading to the oxidative activation of PM CCK1R. In LS, miniSOG-generated ^1^O_2_ may oxidize CCK1R trafficked to LS via the endocytosis pathway, but a partial degradation of CCK1R by LS proteases might hinder the antagonist effect. GEPP: genetically-encoded protein photosensitizers; CCK1R: cholecystokinin 1 receptors; PLC: phospholipase C; IP_3_: inositol-1,4,5-trisphosphate; IP_3_R: inositol-1,4,5-trisphosphate receptors; PM: plasma membrane; MT: mitochondria; and LS: lysosomes.

Other than the plasma membrane expression of miniSOG, mitochondrial (MT) or lysosomal (LS) miniSOG were found to photodynamically trigger persistent calcium oscillations similarly (Figure 3). LED irradiation (450 nm, 85 mW·cm^−2^, 1.5 min)-induced calcium oscillations were inhibited by CCK1R antagonist devazepide 2 nM significantly in miniSOG_PM_-CHO-K1 cells, slightly in miniSOG_MT_-AR4-2J cells, but not at all in miniSOG_LS_-AR4-2J cells (Figure 4), although LED light irradiation induced-calcium oscillations were significantly inhibited by devazepide 10 nM in miniSOG_MT_-AR4-2J cells (Figure 4). The calcium oscillations elicited by miniSOG photodynamic action at the plasma membrane and mitochondria were all likely due to CCK1R activation; at lysosomes, it could be due to CCK1R activation, but in lysosomes, activated CCK1R may not be inhibited by an antagonist, possibly due to the partial proteolysis of the receptor protein (Figure 5). We believe that miniSOG_MT_ photodynamic CCK1R activation might be due to ^1^O_2_ diffusion from the mitochondria to the plasma membrane at mitochondrion-plasma membrane contact sites, which have been found to be widely present [75,76,77]. Such mitochondrion-plasma membrane contact sites may be tethered by proteins such as Num-1 [75] and are nanometers across in size [78]; therefore, miniSOG_MT_ photodynamically generated ^1^O_2_ with a diffusion distance of several tens of nanometers [43] could easily diffuse to the plasma membrane to oxidatively activate CCK1R in miniSOG_MT_-AR4-2J cells. 

The lysosomal accumulation of endocytosized CCK1R and partial CCK1R degradation [62,79,80,81] could possibly account for the miniSOG_LS_ photodynamic CCK1R activation and the little inhibition afforded by CCK1R antagonist devazepide in miniSOG_LS_-AR4-2J cells. The insensitivity of lysosomal CCK1R to the antagonist devazepide 2 nM might also be due to limited accessibility of lysosomal CCK1R to extracellularly added devazepide 2 nM. Such a reduced sensitivity of intracellular GPCR to ligands was noted before for the nuclear membrane GPCR in cardiomyocytes, for example [82]. 

Although the emphasis in the present work was the subcellular localization of miniSOG expression and therefore of subcellular ^1^O_2_ generation, due to its limited lifetime of 1 µs [83,84,85] the effective diffusion distance of ^1^O_2_ in the cellular milieu has been suggested to be in the tens of nanometers or more (20–150 nm) [43,52,83,86,87,88]. Therefore, photodynamically generated ^1^O_2_ could diffuse from the plasma membrane (PM), mitochondrial (MT), or lysosomal (LS) membranes as the origin to within a circle with a radius of 20–150 nm. 

It may be noted that there is abundant evidence for GPCR expression, localization, and function at nuclear [89], mitochondrial [90,91], or other intracellular membranes, such as melanosomal membranes [92], in addition to GPCR transport from the endoplasmic reticulum (ER) to the Golgi apparatus, transport vesicles, PM, and then from the PM by endocytosis to endosomes/lysosomes [61,93,94]. Cardiomyocytes, for example, are endowed with multiple nuclear-membrane GPCR of the A class, such as α1-, β1-, β3-adrenergic receptors, and AT1R and AT2R angiotensin receptors [95,96,97]. The limited diffusion distance of ^1^O_2_ could well help to further investigate GPCR functions at these intracellular sites. Photodynamic GPCR activation/modulation might offer distinct advantages over conventional receptor pharmacology in that no ligand is needed for photodynamic activation after GEPP (KillerRed, miniSOG, miniSOG2, SOPP, Mr4511^C71G^, and DsFbFP) expression at defined intracellular sites. Only light irradiation is required to permanently activate the intracellular GPCR. The limited diffusion distance of photodynamically generated ^1^O_2_ (20–150 nm) may ensure spatial precision and specificity. 

In the present work, all GEPP examined were found to elicit persistent calcium oscillations (i.e., permanent CCK1R activation) photodynamically, either from the PM, MT, or LS. We have previously found that the SALPC photodynamic activation of CCK1R in rat pancreatic acini involved the near quantitative transformation of the CCK1R protein dimer to the monomer [98]. In addition, CCK-induced CCK1R monomerization in purified membrane proteins under sub-threshold SALPC photodynamic action closely followed the CCK dose response curve for amylase secretion in intact rat pancreatic acini, especially in the low, physiological range of CCK concentrations [98]. It would be interesting to see whether GEPP photodynamic CCK1R activation would also involve receptor monomerization. Future works should help to identify the essential structural motif(s) for the permanent ^1^O_2_ activation of CCK1R. 

## 5. Conclusions

In conclusion, representative GEPP (KillerRed, miniSOG, miniSOG2, SOPP, Mr4511^C71G^, and DsFbFP) reported in the literature were found to photodynamically activate the endogenous CCK1R in AR4-2J cells after plasma membrane expression. The miniSOG expression at intracellular sites was also found to induce persistent calcium oscillations or CCK1R activation (Figure 5). The present work provides an effective means to activate CCK1R photodynamically, with the potential for in vivo applications in peripheral physiology and in central nervous system functional studies. Photodynamic activation might also prove suitable for the study of intracellular GPCR, which will involve no ligand additions in the extracellular fluid, therefore overcoming the diffusion barrier imposed by the plasma membrane and without the need for long-distance ligand diffusion through the cytosol. The present work might imply that it is now possible to examine the functional status of the CCK1R protein at different maturation and proteolysis stages in the protein trafficking (ER → Golgi apparatus → transport vesicles → PM) and degradation (endosomes, lysosomes, and proteosomes) pathways.

## Appendix A

**Table A1:** Genetically encoded protein photosensitizers (GEPP).

Photosensitizer	No. AA	Chromophore	λ_ex_ (nm)	λ_em_ (nm)	ϕ^1^O_2_	ϕ_fluo_
**KillerRed**	239	QYG	585	610	0.008	0.25
**SuperNova** **GreenSuperNova**	239 239	QYG QWG	579 440	610 510	0.022 ND	0.30 0.23
**KillerOrange**	239	QWG	512	550	ND	0.42
**TagRFP**	237	MYG	555	584	0.004	0.48
**miniSOG**	106	FMN	448	500	0.03	0.45
**AsLOV2**	175	FMN	448	492	ND	ND
**miniSOG2**	106	FMN	430	503	ND	ND
**Mr4511^C71S/G^** **Pp2FbFP^L30M^** **DsFbFP** **EcFbFP** **CreiLOV**	164 148 138 135 119	FMN FMN FMN FMN FMN	450 449 449 448 449	495 495 498 496 497	0.17/0.19 0.09 0.33 0.07 0.04	ND 0.25 0.35 0.44 0.32
**miniSOG^Q103L/V^**	106	FMN	440	487	0.25/0.39	0.43
**SOPP2/3** **phiSOG** **phiSOG^Q103V^**	106 218 218	FMN FMN FMN	440 449 444	487 498 497	0.51/0.61 0.02 0.15	0.41/0.39 0.36 0.35

Note: No. AA: number of amino acid residues; λ_ex_: maximal excitation wavelength; λ_em_: maximal emission wavelength; ϕ^1^O_2_: quantum yield of ^1^O_2_ generation; ϕ_fluo_: fluorescence quantum yield; (−): generate only O_2_^−.^; and ND: not determined. SOPP = miniSOG^Q103L^.

**Table A2:** Peptide sequence of KillerRed, miniSOG, miniSOG2, SOPP, Mr4511^C71G^, and DsFbFP.

Plasmid Name	Backbone	Insert/Replacement
*pKillerRed_PM_*	*pKillerRed_PM_*	KillerRed
MLCCMRRTKQVEKNDEDQKI MGSEGGPALFQSDMTFKIFIDGEVNGQKFTIVADGSSKFPHGDFNVHAVCETGKLPMSWKPICHLIQYGEPFFARYPDGISHFAQECFPEGLSIDRTVRFENDGTMTSHHTYELDDTCVVSRITVNCDGFQPDGPIMRDQLVDILPNETHMFPHGPNAVRQLAFIGFTTADGGLMMGHFDSKMTFNGSRAIEIPGPHFVTIITKQMRDTSDKRDHVCQREVAYAHSVPRITSAIGSDED		
*pminiSOG_PM_*	*pKillerRed_PM_*	miniSOG
MLCCMRRTKQVEKNDEDQKI MEKSFVITDPRLPDNPIIFASDGFLELTEYSREEILGRNGRFLQGPETDQATVQKIRDAIRDQREITVQLINYTKSGKKFWNLLHLQPMRDQKGELQYFIGVQLDG		
*pminiSOG_MT_*	*pminiSOG_MT_*	MT localization sequence
MSVLTPLLLRGLTGSARRLPVPRAKIHSLGDPPVAT MEKSFVITDPRLPDNPIIFASDGFLELTEYSREEILGRNGRFLQGPETDQATVQKIRDAIRDQREITVQLINYTKSGKKFWNLLHLQPMRDQKGELQYFIGVQLDG		
*pminiSOG_LS_*	*pminiSOG_LS_*	LS localization sequence
KGQGSMDEGTADERAPLIRT MEKSFVITDPRLPDNPIIFASDGFLELTEYSREEILGRNGRFLQGPETDQATVQKIRDAIRDQREITVQLINYTKSGKKFWNLLHLQPMRDQKGELQYFIGVQLDG		
*pminiSOG2_PM_*	*pminiSOG_PM_*	miniSOG2
MLCCMRRTKQVEKNDEDQKI MEKSFVITDPRLPDNPIIFASDSFLELTEYSREEILGRNPRFLRGPETDQATVQKIHDAIRDQREITVQLINYTKSGKKFWNLFRLQPIRDQKGELQYFIGVQLDG		
*pSOPP_PM_*	*pminiSOG_PM_*	SOPP
MLCCMRRTKQVEKNDEDQKI MEKSFVITDPRLPDNPIIFASDGFLELTEYSREEILGRNGRFLQGPETDQATVQKIRDAIRDQREITVQLINYTKSGKKFWNLLHLQPMRDQKGELQYFIGVLLDG		
*pMr4511^C71G^_PM_*	*pminiSOG_PM_*	Mr4511^C71G^
MLCCMRRTKQVEKNDEDQKI METGGTATSHVPDELKAESHRGDPFAAAVRATRMPMIITDPAQHDNPIVFVNDAFLKLTGYTRMEVVGRNGRFLQGPDTEAAAVDRLRAAIRREEDIRVDLLNYRKDGSTFQNALYVGPVRDEAGRVVYFFASQLDVSEHYALTAEIERLKAALAEAEAKLAAR		
*pDsFbFP_PM_*	*pminiSOG_PM_*	DsFbFP
MLCCMRRTKQVEKNDEDQKI MRRHYRDLIRNTPMPDTPQDIADLRALLDEDEAEMSVVFSDPSQPDNPMIYVSDAFLVQTGYTLEEVLGRNARFLQGPDTNPHAVEAIRQGLKAETRFTIDILNYRKDGSAFVNRLRIRPIYDPEGNLMFFAGAQNPVLE		

The color code corresponds to the translated sequences of: the plasma membrane localization sequence, KillerRed, miniSOG, miniSOG2, SOPP/miniSOG^Q103L^, Mr4511^C71G^, DsFbFP, and the mitochondrial/lysosomal localization sequence. Note that the Evrogen *pKillerRed* plasmid does not encode the initial two residues MG.
